# Genomic imprinting does not reduce the dosage of UBE3A in neurons

**DOI:** 10.1186/s13072-017-0134-4

**Published:** 2017-05-15

**Authors:** Paul R. Hillman, Sarah G. B. Christian, Ryan Doan, Noah D. Cohen, Kranti Konganti, Kory Douglas, Xu Wang, Paul B. Samollow, Scott V. Dindot

**Affiliations:** 1 Department of Veterinary Pathobiology, College of Veterinary Medicine and Biomedical Sciences, Texas A&M University, College Station, TX 77845 USA; 2 Department of Molecular and Cellular Medicine, College of Medicine, Texas A&M Health Science Center, College Station, TX 77845 USA; 3 Interdisciplinary Genetics Program, College of Agriculture and Life Sciences, Texas A&M University, College Station, TX 77845 USA; 4 Department of Large Animal Clinical Sciences, College of Veterinary Medicine and Biomedical Sciences, Texas A&M University, College Station, TX USA; 5 Institute for Genome Science and Society, Texas A&M University, College Station, TX 77845 USA; 6 Department of Veterinary Integrative Biosciences, College of Veterinary Medicine and Biomedical Sciences, Texas A&M University, College Station, TX 77843 USA; 7 Department of Molecular Biology and Genetics, Cornell University, Ithaca, NY 14853 USA; 8 Department of Veterinary Pathobiology, College of Veterinary Medicine and Biomedical Sciences, Texas A&M University, 4467 TAMU, College Station, TX 77843 USA

**Keywords:** *Ube3a*, Genomic imprinting, Dosage compensation, Angelman syndrome, *Ube3a* antisense, Evolution

## Abstract

**Background:**

The ubiquitin protein E3A ligase gene (*UBE3A*) gene is imprinted with maternal-specific expression in neurons and biallelically expressed in all other cell types. Both loss-of-function and gain-of-function mutations affecting the dosage of UBE3A are associated with several neurodevelopmental syndromes and psychological conditions, suggesting that UBE3A is dosage-sensitive in the brain. The observation that loss of imprinting increases the dosage of UBE3A in brain further suggests that inactivation of the paternal *UBE3A* allele evolved as a dosage-regulating mechanism. To test this hypothesis, we examined *UBE3A* transcript and protein levels among cells, tissues, and species with different imprinting states of *UBE3A*.

**Results:**

Overall, we found no correlation between the imprinting status and dosage of UBE3A. Importantly, we found that maternal Ube3a protein levels increase in step with decreasing paternal Ube3a protein levels during neurogenesis in mouse, fully compensating for loss of expression of the paternal *Ube3a* allele in neurons.

**Conclusions:**

Based on our findings, we propose that imprinting of *UBE3A* does not function to reduce the dosage of *UBE3A* in neurons but rather to regulate some other, as yet unknown, aspect of gene expression or protein function.

**Electronic supplementary material:**

The online version of this article (doi:10.1186/s13072-017-0134-4) contains supplementary material, which is available to authorized users.

## Background

 Genomic imprinting is a rare epigenetic phenomenon that leads to the differential expression of paternally and maternally derived alleles of a gene in a parent-of-origin dependent manner [50, 54]. It has been documented only in therian mammals and flowering plants and only at a few loci in mammals, of which fewer than half are imprinted in both mouse and human [1, 4, 8, 39, 42, 50, 52]. Several theories have been proposed to explain the evolution and function of genomic imprinting (e.g., dosage regulation, complementation, parental conflict/kinship, host defense, maternal time-bomb, and developmental plasticity models), but there is currently no unifying theory that can explain the conservation of genomic imprinting across taxa [2, 17–19, 35, 50, 51, 58, 59]. Thus, apart from downregulating or silencing the expression of one allele, the functional significance of imprinting is largely unknown. Nevertheless, imprinting is believed to be an important regulatory mechanism, inasmuch as almost all recognized imprinting abnormalities are associated with pathological states [8, 49].

 The ubiquitin protein E3A ligase gene (*UBE3A*) is located at the distal end of a cluster of imprinted genes on human chromosome 15q11-q13 and a homologous region on mouse chromosome 7C. In neurons of the central nervous system (CNS), *UBE3A* is imprinted with maternal-allelic expression, whereas in all other cell types, it is expressed from both alleles [11, 43, 62]. Imprinting of *UBE3A* is regulated by expression of the paternally expressed *UBE3A* antisense transcript (*UBE3A*-*AS*), which comprises the 3′ end of a long polycistronic transcription unit (PTU) containing multiple clusters of C/D box small nucleolar RNAs (snoRNA) and the *SNRPN*-*SNURF* bicistronic transcript [7, 29, 30, 44]. In both mouse and human, expression of *Ube3a*-*AS*/*UBE3A*-*AS in cis* is both necessary and sufficient to silence expression of the paternal *Ube3a*/*UBE3A* allele [ [31], [33] ], which, at least in mouse, appears to occur by inhibiting transcriptional elongation, giving rise to a paternally expressed, 5′-truncated transcript of unknown function [33, 38].

The *UBE3A* gene is highly conserved among vertebrate and invertebrate species [6, 9, 21, 22, 61]; however, imprinting of *UBE3A* is believed to have evolved in the common ancestor of eutherian mammals after divergence from the metatherian (marsupials) lineage, as studies to date show that *UBE3A* is biallelically expressed in tammar wallaby, platypus, chicken, and fruit-fly brain, and that there is no orthologous imprinted region detectable in marsupials or monotreme (prototherian) mammals [9, 21, 41], suggesting that imprinting of *UBE3A* in neurons is somehow advantageous to biallelic expression in eutherian species. The snoRNAs located in 15q11-q13, which serve as the precursors of *Ube3a*-*AS*/*UBE3A*-*AS*, are also eutherian-specific and appear to have rapidly evolved in a lineage-specific manner [64]; however, the relationship, if any, between these transcripts or the evolution of this region and imprinting of *Ube3a*/*UBE3A* is currently unknown.

Mutations or epimutations affecting the expression or function of *UBE3A* are associated with several neurodevelopmental disorders and psychological conditions. Loss-of-function or loss-of-expression of the maternally inherited *UBE3A* allele causes Angelman syndrome (AS), which presents with intellectual disability, ataxia, epilepsy, sleep disorders, and an atypical ‘happy’ disposition [26, 32, 60]. Conversely, maternally derived duplications of 15q11-q13 cause dup15q syndrome—a neurodevelopmental disorder distinctly different from AS—involving intellectual disability, ataxia, speech impairment, epilepsy, and autism spectrum disorder (ASD) [16, 20, 36, 40, 47]. Although dup15q syndrome is a contiguous gene disorder, overexpression of *UBE3A* is believed to be the principal pathological mechanism underlying the syndrome, as *UBE3A* is the only maternally expressed gene located within the duplication and as the severity of the condition correlates with the number of copies and expression levels of *UBE3A* [47]. There are also reports of *UBE3A* gain-of-function mutations (e.g., biochemical and genetic) in individuals with ASD and other psychological conditions [37, 63]. Paternally inherited deletions of 15q11-q13, namely those involving the C/D box snoRNA *SNORD116*, result in Prader–Willi syndrome (PWS), which is characterized by dysregulated hunger and satiety patterns, abnormal thermoregulation, sleep disorders, and behavioral issues [45].

The notion that imprinting of *UBE3A* evolved as a dosage-regulating mechanism stems from the role of *UBE3A* in AS and dup15q syndromes and from observations showing that loss of *Ube3a*-*AS* reactivates paternal *Ube3a* expression, leading to an increase in Ube3a protein levels in the brain [5, 7, 13, 34]. In the present study, we compared *UBE3A* expression levels (RNA transcript and protein) between cells, tissues, and species with different imprinting states of *UBE3A*. We also examined parental Ube3a protein levels during the acquisition of the imprint in neurons. Overall, our findings show that the dosage of Ube3a/UBE3A is relatively constant regardless of its imprinting status, suggesting that imprinting does not function to regulate the dosage of Ube3a/UBE3A in neurons.

## Results

### *Ube3a*/*UBE3A* is highly expressed from the maternal allele in the CNS

**Fig. 1:**
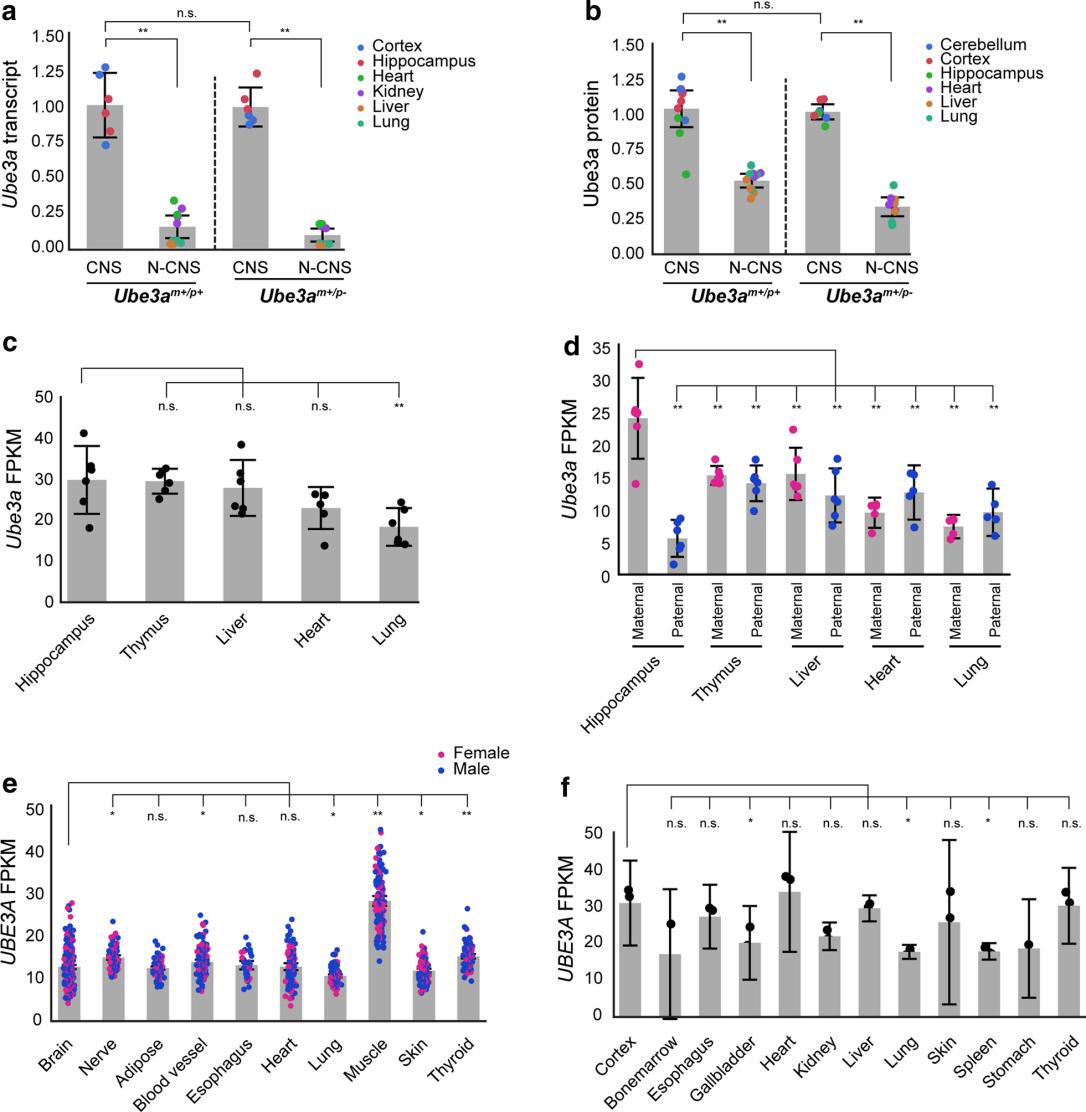
*Ube3a*/*UBE3A* is highly expressed in mouse and human CNS tissues. **a** *Ube3a* transcript levels in adult *Ube3a* ^m+/p+^ (*n* = 4) and *Ube3a* ^m+/p−^ (*n* = 3) CNS (cortex, hippocampus) and non-CNS (N-CNS; heart, kidney, liver, and lung) tissues. Levels are shown as the ratio of expression in tissues relative to heart. **b** Ube3a protein levels in adult *Ube3a* ^m+/p+^ (*n* = 4) and *Ube3a* ^m+/p−^ (*n* = 3) CNS (cerebellum, cortex, hippocampus) and non-CNS (heart, liver, and lung). Levels are shown as the ratio of expression in tissues relative to heart. **c** Normalized FPKM values of total *Ube3a* transcripts and **d** maternal and paternal *Ube3a* transcripts in B6D2F1 mouse hippocampus, thymus, liver, lung, and heart (*n* = 6). **e, f** Normalized FPKM values of *UBE3A* transcripts in adult human tissues (GTEx: *n* ≥ 30; Fagerberg et al.: *n* = 3). *Abbreviations* n.s., not significant; *p < 0.05; **p < 0.001. Individual data points provided with mean (*gray bar chart*) and 95% confidence intervals

To determine whether imprinting of *Ube3a* in neurons of the mouse CNS reduces the dosage of *Ube3a* relative to other tissues where *Ube3a* is biallelically expressed (non-CNS), we compared the steady-state levels of *Ube3a* RNA (hereafter referred to as transcript) and Ube3a protein among tissues in adult wild-type mice. To estimate the relative expression levels of the maternal *Ube3a* allele, we also examined mice with a paternally inherited mutation in the *Ube3a* gene [23]. For both *Ube3a* ^m+/p+^ and *Ube3a* ^m+/p−^ mice, *Ube3a* transcript levels were significantly higher in CNS (cortex and hippocampus) than in non-CNS (heart, kidney, liver, and lung) tissues [*Ube3a* ^m+/p+^: *F*(1, 15.8) = 55.6, *p* < 0.0001; *Ube3a* ^m+/p−:^ *F*(1, 14) = 338.6, *p* < 0.0001 (Fig. 1a)]. Likewise, Ube3a protein levels in both *Ube3a* ^m+/p+^ and *Ube3a* ^m+/p−^ mice were significantly higher in CNS (cerebellum, cortex, hippocampus) than in non-CNS (heart, liver, and lung) tissues [*Ube3a* ^m+/p+^: *F*(1, 19) = 22.8, *p* < 0.0001; *Ube3a* ^m+/p−:^ *F*(1, 14) = 118.5, *p* < 0.001 (Fig. 1b)]. Neither *Ube3a* RNA or Ube3a protein levels were significantly different in the CNS of *Ube3a* ^m+/p+^ and *Ube3a* ^m+/p−^ mice (RNA: *t* = 0.9, *p* = 0.8; protein: CNS, *t* = 2.1, *p* = 0.2), indicating that the relatively high level of *Ube3a* expression in mouse CNS is primarily attributable to expression of the maternal allele.

To confirm that the maternal *Ube3a* allele is highly expressed in the CNS, we used RNA-seq data to compare *Ube3a* transcript levels expressed from each parental allele within and among CNS (hippocampus) and non-CNS (heart, liver, lung, and thymus) tissues in adult mice [25]. Total *Ube3a* transcript levels (i.e., those expressed by both maternal and paternal alleles) were significantly different among the tissues [*F*(4, 41.8) = 8.3, *p* < 0.0001], with slightly higher, albeit not significant, *Ube3a* transcript levels in hippocampus compared to those in liver (*t* = 0.8, *p* = 0.9) and thymus (*t* = 0.1, *p* = 0.9) and significantly higher transcript levels than those in heart (*t* = 2.9, *p* = 0.02) and lung [*t* = 4.7, *p* < 0.0001 (Fig. 1c)]. Importantly, we found that maternal *Ube3a* transcript levels in hippocampus were significantly higher than those expressed from either allele in all the other tissues, whereas paternal *Ube3a* transcript levels in hippocampus were lower than those expressed from either allele in all the other tissues, with significantly lower levels relative to hippocampus, thymus, liver, and heart (paternal allele) (Fig. 1d and Additional file 1), confirming that the relatively high levels of *Ube3a* expression in the CNS arise from the maternal allele.

To compare *UBE3A* expression levels between CNS and non-CNS tissues in human, we analyzed *UBE3A* transcript levels in 10 tissues (*n* ≥ 30) using RNA-seq data generated by the GTEx consortium [10] and in 12 tissues (*n* = 3) using RNA-seq data generated by Fagerberg et al. [12]. In the GTEx data set, *UBE3A* transcript levels were significantly different among the tissues [*F*(9, 1129.8) = 262.5, *p* < 0.0001], with no significant effect of sex [*F*(1, 186.6) = 0.2, *p* = 0.6] or significant interaction between tissue and sex [*F*(9, 1129.8] = 0.8, *p* = 0.6). Relative to brain, *UBE3A* transcript levels were similar to those in heart, adipose tissue, and esophagus, significantly higher than those in lung and skin, but significantly lower than those in blood vessel, muscle, thyroid, and nerve (tibial) (Fig. 1e and Additional file 2). In the Fagerberg data set, *UBE3A* transcript levels were also significantly different among the tissues [*F*(11, 14) = 7.5, *p* < 0.0004]. Relative to cortex, *UBE3A* transcript levels were similar to those in bone marrow, esophagus, heart, kidney, liver, skin, and thyroid, and significantly higher than those in gallbladder, lung, spleen, and stomach (Fig. 1f and Additional file 3).

Taken together, these findings show that in both mouse and human cells, expression levels of *Ube3a*/*UBE3A* in CNS are generally equal to or higher than those in non-CNS tissues, despite the different imprinting states.

### Maternal *Ube3a* compensates for loss of paternal *Ube3a* expression during neurogenesis

**Fig. 2:**
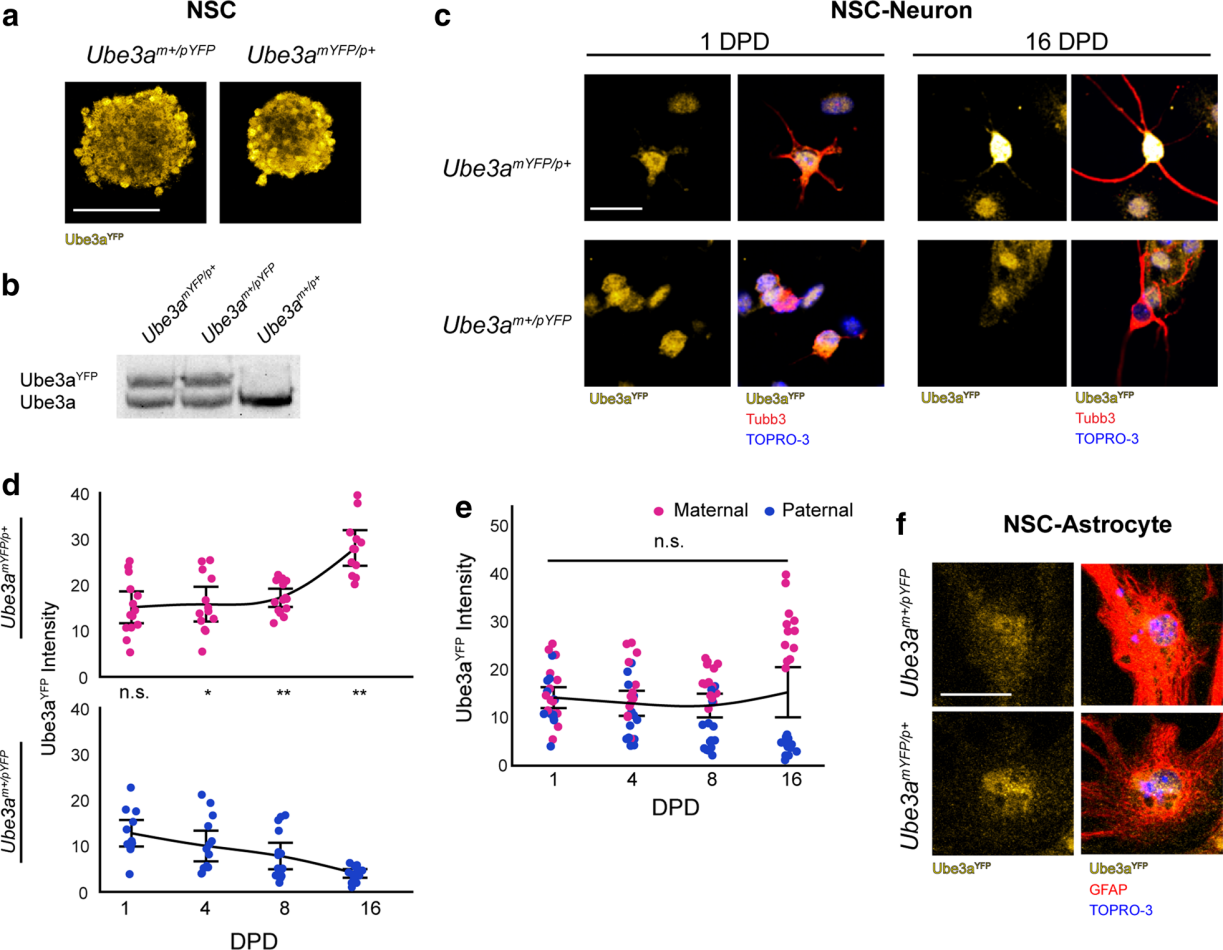
Maternal *Ube3a* compensates for loss of paternal *Ube3a* expression during neurogenesis. **a** Immunofluorescence images of primary neurospheres derived from the hippocampal formation of prenatal *Ube3a* ^*m*+/*pYFP*^ and *Ube3a* ^*mYFP*/*p*+^ mice. *Scale bar* = 100 µm. **b** Western blot analysis of Ube3a and Ube3a^YFP^ protein in NSC derived from *Ube3a* ^*m*+/*pYFP*^, *Ube3a* ^*mYFP*/*p*+^ and wild-type mice. **c** Immunofluorescence images of *Ube3a* ^*m*+/*pYFP*^ and *Ube3a* ^*mYFP*/*p*+^ NSC-derived neurons at 1 and 16 days post-differentiation (DPD; *scale bar* = 25 µm). **d** Ube3a^YFP^ intensity values of paternal Ube3a^YFP^ and maternal Ube3a^YFP^ protein levels in primary neurons at 1 DPD (*Ube3a* ^*m*+/*pYFP*^: *n* = 13; *Ube3a* ^*mYFP*/*p*+^ *n* = 14), 4 DPD (*Ube3a* ^*m*+/*pYFP*^: *n* = 14; *Ube3a* ^*mYFP*/*p*+^: *n* = 13), 8 DPD (*Ube3a* ^*m*+/*pYFP*^: *n* = 15; *Ube3a* ^*mYFP*/*p*+^: *n* = 15), and 16 DPD (*Ube3a* ^*m*+/*pYFP*^: *n* = 14; *Ube3a* ^*mYFP*/*p*+^: *n* = 13). Ratios normalized relative to total Ube3a^YFP^ protein levels at 1 DPD. **e** Total Ube3a^YFP^ protein levels in neurons during neuronal development. **f** Normalized paternal Ube3a^YFP^ and maternal Ube3a^YFP^ protein levels in *Ube3a* ^*m*+/*pYFP*^ and *Ube3a* ^*mYFP*/*p*+^ NSC-derived astrocytes at 16 DPD (*Ube3a* ^*m*+/*pYFP*^: *n* = 15; *Ube3a* ^*mYFP*/*p*+^: *n* = 14). *Abbreviations* TOPRO-3, nuclear stain; n.s., not significant; *p < 0.05; **p < 0.001. Individual data points provided with 95% confidence intervals

Our findings that the maternal *Ube3a* allele is highly expressed in brain prompted us to examine the expression of each parental *Ube3a* allele during the acquisition of the imprint in neurons. Using the *Ube3a* ^*YFP*^ reporter mouse model [11], we compared paternal Ube3a^YFP^ (*Ube3a* ^*m*+/*pYFP*^) and maternal Ube3a^YFP^ (*Ube3a* ^*mYFP*/*p*+^) protein levels in neural stem/progenitor cells (NSC) and in NSC-derived neurons over the course of 16 days of differentiation in vitro. In NSC cultures, maternal and paternal Ube3a^YFP^ protein levels were approximately equal [maternal/paternal ratio = 48.3:51.7; *F*(1, 2) = 0.8, *p* = 0.5 (Fig. 2a, b)], which was not affected by the parent-of-origin of the Ube3a^YFP^ reporter allele [*F*(1, 2) = 1.31, *p* = 0.4] or number of passages in culture (data not shown). In NSC-derived neurons, paternal and maternal Ube3a^YFP^ protein levels were approximately equal at 1 day post-differentiation (DPD; *t* = 0.9, *p* = 0.4); however, at 4, 8, and 16 DPD, maternal Ube3a^YFP^ protein levels were significantly higher than paternal Ube3a^YFP^ protein levels (4 DPD: *t* = 2.9, *p* = 0.004; 8 DPD: *t* = 5.7, *p* < 0.0001; and 16 DPD: *t* = 11.8, *p* < 0.0001), with the protein levels produced by each parental allele diverging at a similar rate (slope: maternal = 0.76, paternal = −0.56; Fig. 2c, d). As a result, total Ube3a^YFP^ protein levels in neurons remained relatively constant during the 16 days of differentiation [*F*(3, 3) = 1.3, *p* = 0.3 (Fig. 2e)], demonstrating that the maternal *Ube3a* allele compensates for loss of expression of the paternal *Ube3a* allele during the acquisition of the imprint in neurons. In contrast, paternal and maternal Ube3a^YFP^ protein levels were approximately equal in NSC-derived astrocytes at 16 DPD [*t* = 0.3, *p* = 0.8; maternal/paternal ratio = 45.4:54.6 (Fig. 2f)]. Taken together, these findings indicate that imprinting of *Ube3a* is initiated upon neuronal differentiation and that the maternal *Ube3a* allele fully compensates for loss of expression of the paternal allele in neurons.

### Ube3a/UBE3A protein levels in the mouse and opossum brain do not correlate with their imprinting status

The *UBE3A* gene is highly conserved among vertebrates, but its specific location within the AS-PWS imprinted region in eutherian (placental) mammals suggests that imprinting of *UBE3A* evolved subsequent to divergence of the eutherian and metatherian (marsupial) lineages. Expression data from unspecified tissues of tammar wallaby (*Macropus eugenii*) and platypus (*Ornithorhynchus anatinus*) brain show that *UBE3A* is not imprinted in these species, consistent with this evolutionary scenario [41]. If genomic imprinting of *UBE3A* arose in the common ancestor of modern eutherian mammals as an epigenetic mechanism to reduce the dosage of UBE3A in neurons, we should expect levels of UBE3A in the CNS of eutherian mammals to be reduced relative to that in other (non-CNS) cell types, but have no expectation of a similar pattern of relative reduction in CNS cells of non-eutherian mammals. To test this hypothesis, we contrasted Ube3a/UBE3A protein levels in non-CNS tissues and cortex, within and between mouse and the gray, short-tailed opossum (*Monodelphis domestica*), a metatherian mammal lacking an orthologous AS-PWS region. We first confirmed the non-imprinted imprinting status of *UBE3A* in opossum cortex using RNA-seq. We next used western blot analysis of UBE3A protein levels to obtain measures of relative expression among opossum tissues and normalized, absolute expression between mouse and opossum cortex.

**Fig. 3:**
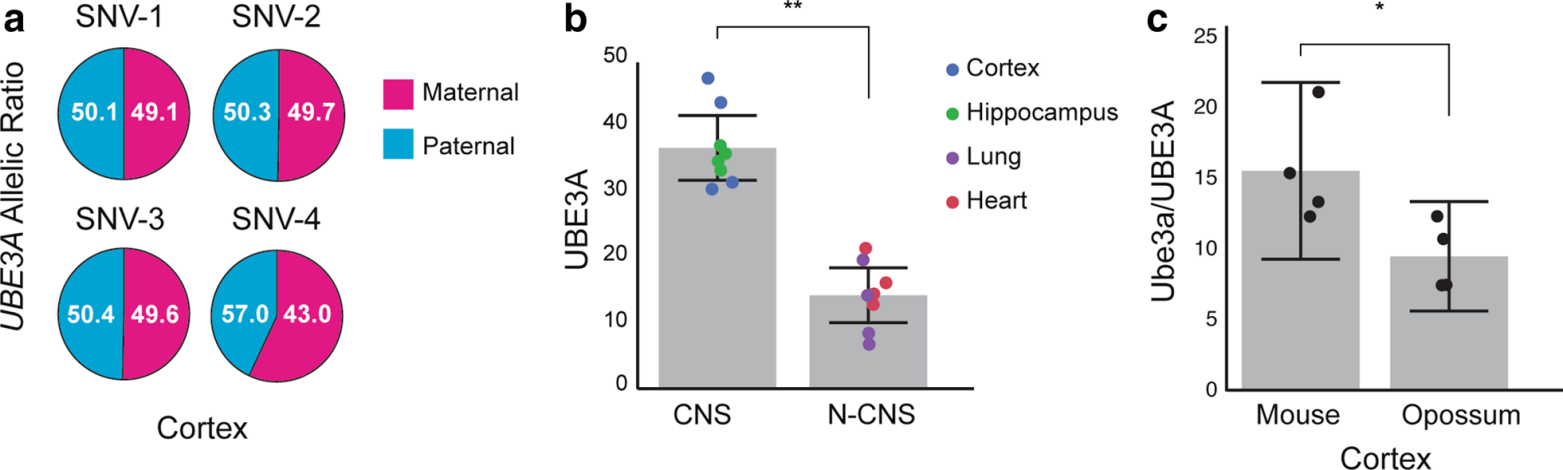
Ube3a protein levels are higher mouse and opossum brain despite different imprinting states of *Ube3a*/*UBE3A*. **a** *UBE3A* allelic ratio expressed in adult opossum cortex (*n* = 4) estimated from four single-nucleotide variants (SNV). **b** Normalized UBE3A protein levels in adult opossum CNS (cortex and hippocampus) and non-CNS (N-CNS; heart and lung) tissues (*n* = 4). **c** Normalized UBE3A/Ube3a protein levels in adult mouse (*n* = 4) and opossum (*n* = 4) cortex. *Abbreviations* n.s., not significant; *p < 0.05; **p < 0.001. Individual data points provided with mean (*gray bar chart*) and 95% confidence intervals

Analysis of RNA-seq data derived from opossum cortex (*n* = 4) revealed 4 informative SNVs in *UBE3A* that were equally represented from each parental allele [SNV-1: *X* ^2^ = 0.2, *p* = 0.7; SNV-2: *X* ^2^ = 0.02, *p* = 0.9; SNV-3: *X* ^2^ = 0.9, *p* = 0.7; SNV-4: *X* ^2^ = 2.5, *p* = 0.1 (Fig. 3a and Additional file 4)]. Combined, the maternal-to-paternal allelic ratio of *UBE3A* transcripts was 49:51 (95% CI maternal: 46–51, paternal: 51–54), confirming that *UBE3A* is biallelically expressed in opossum brain. Alignment of the mouse, human, and opossum Ube3a/UBE3A amino acid sequences revealed a high percent identity (93–95% identical) among the three species, indicating that opossum *UBE3A* is sufficiently similar to the eutherian protein to enable meaningful comparisons by western blot (Additional file 5). Consistent with our findings in mice, UBE3A protein levels in the opossum CNS (cortex and hippocampus) were significantly higher than those in non-CNS tissues [heart and lung; *F*(1, 11) = 38.6, *p* < 0.001 (Fig. 3b)], suggesting that an increased level of expression in cortex may be a conserved characteristic of all therian mammals, independent of imprinting. Importantly, direct comparisons between mouse and opossum showed significantly higher levels of Ube3a protein in the mouse cortex (*n* = 4) than in the opossum cortex [*t* = 2.6, *p* < 0.05 (Fig. 3c)]. Thus, despite imprinted expression of *Ube3a* in the mouse CNS, Ube3a protein levels in cortex are substantially higher than those produced by biallelic expression in the opossum cortex.

### Imprinting of *Ube3a* during neurogenesis is developmentally regulated

Given our findings that expression of the paternal *Ube3a* allele gradually decreases during neuronal development in vitro, we decided to examine the developmental timing of the acquisition of the imprint in vivo by examining parental Ube3a^YFP^ protein expression in two neurogenic regions of the adult mouse brain: (1) the subgranular zone of the dentate gyrus (SGZ) and (2) the subventricular zone of the lateral ventricles (SVZ) and rostral migratory stream (RMS). In the SGZ, neural stem cells differentiate into mature granular neurons that integrate into the dentate gyrus through migration to the granular cell layer (GCL), whereas in the SVZ, neural stem cells differentiate while migrating through the RMS to the olfactory bulb, where they differentiate into mature olfactory neurons [28].

**Fig. 4:**
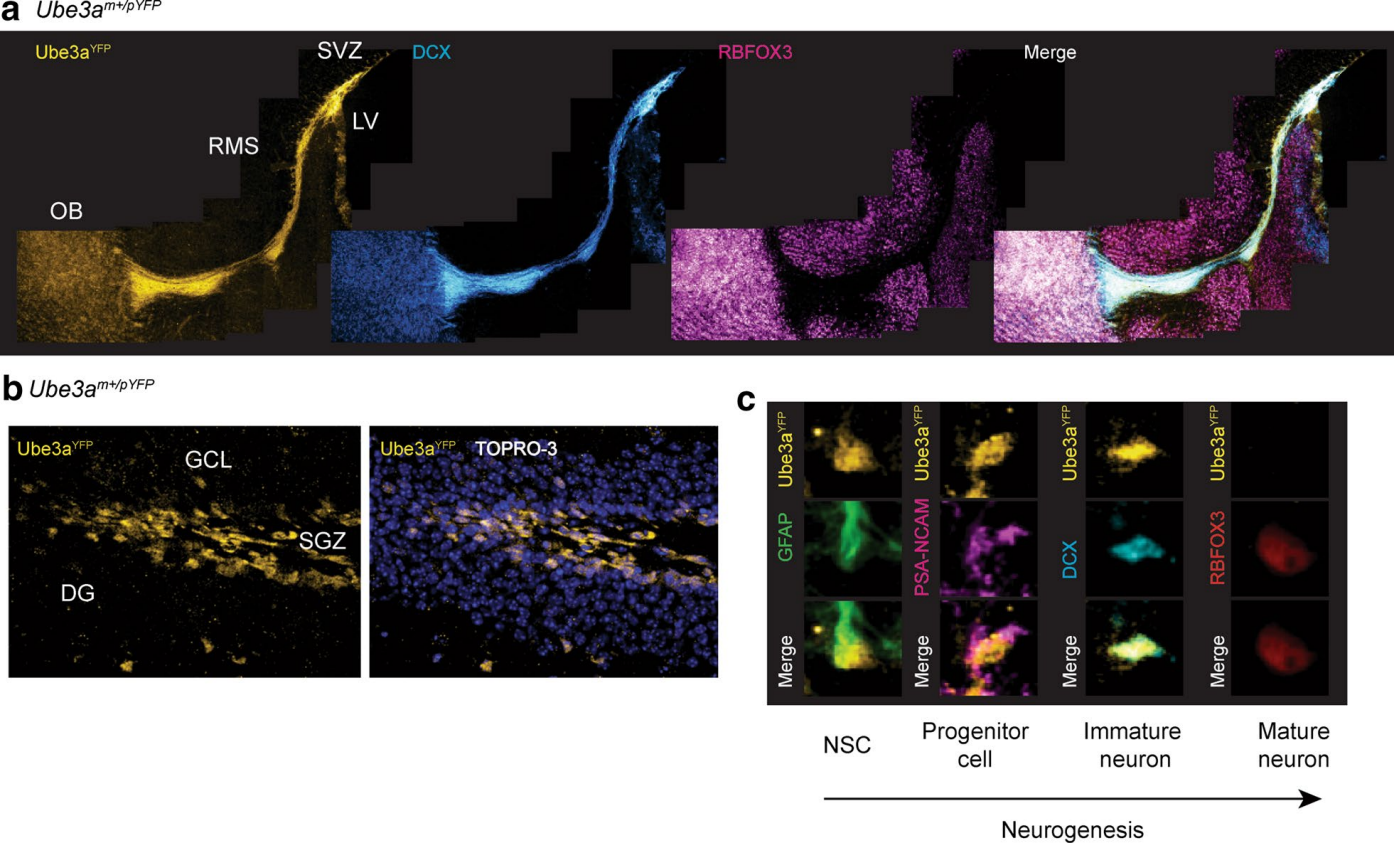
Imprinting of *Ube3a* is developmentally regulated. **a** Immunofluorescence images of paternal Ube3a^YFP^ protein expression in the subventricular zone, rostral migratory stream, and olfactory bulb of *Ube3a* ^*m*+/*pYFP*^ mice. Immature and mature neurons were identified by DCX and RBFOX3 staining, respectively (×10 magnification; *scale bar* = 100 µm). **b** Immunofluorescence images of paternal Ube3a^YFP^ protein expression in the dentate gyrus of *Ube3a* ^*m*+/*pYFP*^ mice (×10 magnification; *scale bar* = 20 µm). **c** Immunofluorescence images of paternal Ube3a^YFP^ in putative neural stem cells (GFAP labeled radial glia), neural progenitor cells (PSA-NCAM), immature neurons (DCX), and mature neurons (RBFOX3) of the subgranular zone (×43 magnification; *scale bar* = 20 µm). *Abbreviations* OB, olfactory bulb; RMS, rostral migratory stream; SVZ, subventricular zone; LV, lateral ventricle; DG, dentate gyrus; GCL, granular cell layer; SGZ, subgranular zone; TOPRO-3, nuclear stain; NSC, neural stem cell

 In the SVZ of adult mice, paternal Ube3a^YFP^ protein was detected in NSC/progenitor cells located along the lateral ventricles and expressing the polysialylated-neural cell adhesion molecule (PSA-NCAM) and nestin (NES) (Additional File 6). In the RMS, paternal Ube3a^YFP^ protein was detected in immature neurons expressing doublecortin (DCX); however, in the olfactory bulb, paternal Ube3a^YFP^ protein was barely detectable in mature neurons expressing the RNA-binding protein, Fox-1 homolog 3 [RBFOX3 (Fig. 4a)]. In the dentate gyrus, paternal Ube3a^YFP^ protein was only detected in the SGZ, where it was present in radial glia (i.e., putative neural stem cells) expressing glial fibrillary acidic protein (GFAP), progenitor cells expressing PSA-NCAM, and immature neurons expressing DCX but not in mature neurons expressing RBFOX3 in the GCL (Fig. 4b, c). In contrast, maternal Ube3a^YFP^ protein was detected in each neurogenic cell type and in mature neurons throughout the CNS (Additional file 6). Taken together, these observations indicate that expression of the paternal *Ube3a* allele is silenced at a specific stage of neurogenesis, preceding the developmental maturation of neurons.

### *Ube3a* is biallelically expressed in myenteric neurons of the PNS

**Fig. 5:**
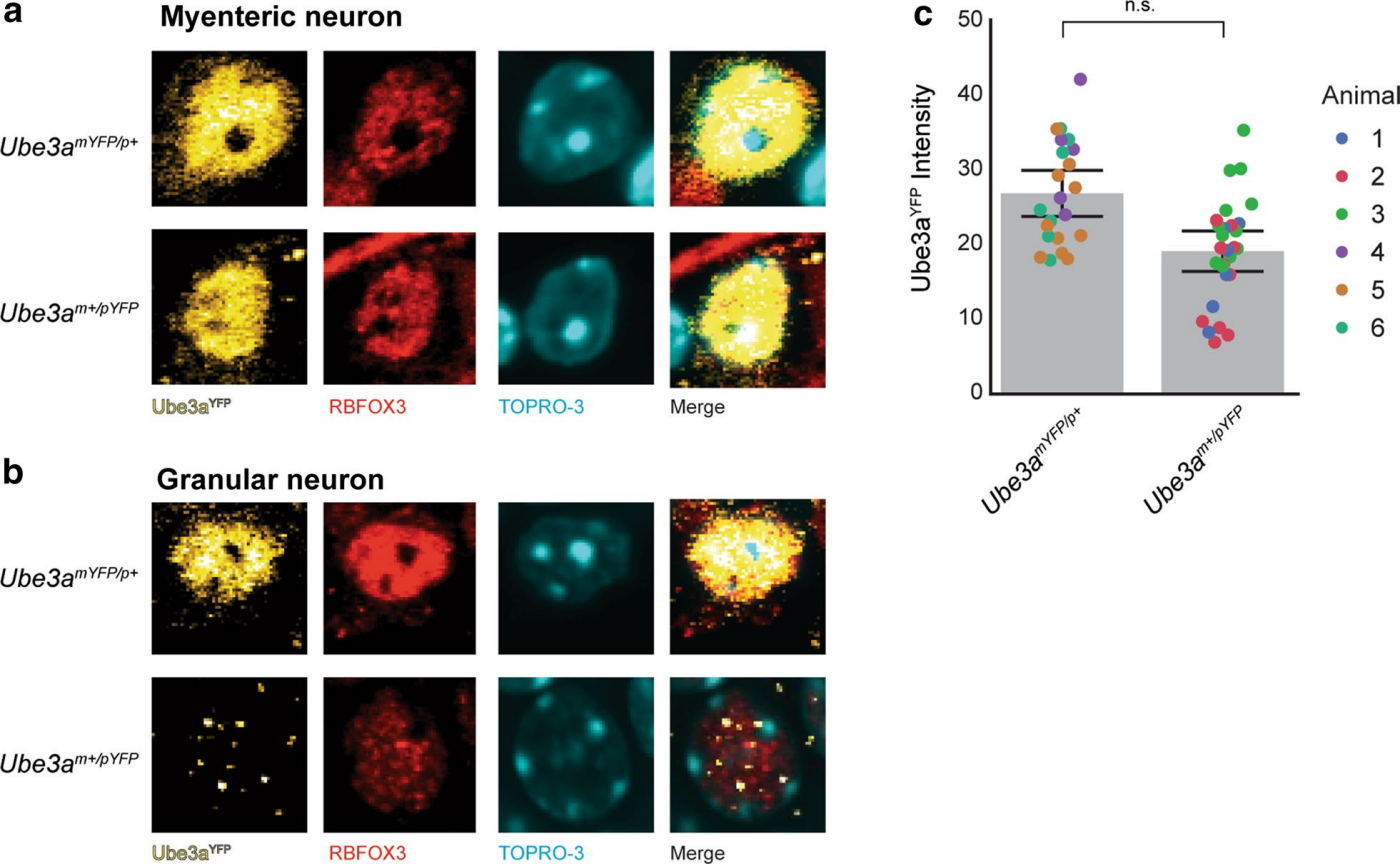
*Ube3a* is biallelically expressed in myenteric neurons of the PNS. **a** Immunofluorescence images of neurons in the myenteric ganglia of the peripheral nervous system of *Ube3a* ^*m*+/*pYFP*^ and *Ube3a* ^*mYFP*/*p*+^ mice. **b** Immunofluorescence images of granular neurons in the dentate gyrus of *Ube3a* ^*m*+/*pYFP*^ and *Ube3a* ^*mYFP*/*p*+^ mice. **c** Normalized intensity values of Ube3a^YFP^ protein levels in myenteric neurons of *Ube3a* ^*m*+/*pYFP*^ and *Ube3a* ^*mYFP*/*p*+^ mice (*n* = 3 per genotype; *Ube3a* ^*m*+/*pYFP*^: *n* = 22 neurons; *Ube3a* ^*mYFP*/*p*+^: *n* = 28). *Abbreviations* TOPRO-3, nuclear stain; n.s., not significant. Individual data points provided with mean (*gray bar chart*) and 95% confidence intervals

Lastly, we asked whether *Ube3a* is also imprinted in neurons of the peripheral nervous system (PNS). To test this hypothesis, we examined parental Ube3a^YFP^ protein levels in myenteric neurons in Auerbach’s ganglia in the colon. In adult *Ube3a* ^*mYFP*/*p*+^ and *Ube3a* ^*m*+/*pYFP*^ mice, maternal Ube3a^YFP^ and paternal Ube3a^YFP^ protein were detected in mature myenteric neurons expressing RBFOX3 (Fig. 5a), in contrast to hippocampal granular neurons in which paternal Ube3a^YFP^ protein was almost undetectable (Fig. 5b). The levels of Ube3a^YFP^ protein expressed from each allele in myenteric neurons, however, was skewed, with higher levels of maternal Ube3a^YFP^ protein albeit not significantly different (maternal/paternal ratio = 60:40; *F*(1, 4.2) = 6.3, *p* = 0.06) (Fig. 5b). Although studies of additional PNS neurons are needed, these findings suggest that imprinting of *Ube3a* is likely specific to neurons of the CNS.

## Discussion

In this study, we examined whether imprinting functions to reduce the dosage of Ube3a/UBE3A (i.e., levels of transcripts and/or protein) in neurons. Our results show that imprinting of the mouse *Ube3a* gene in neurons is achieved through increasing expression of the maternal *Ube3a* allele in step with decreasing expression of the paternal *Ube3a* allele that occurs prior to a specific developmental time point during neurogenesis. These compensating allelic expression states maintain a relatively constant level of Ube3a protein in neurons and relatively high levels of *Ube3a* RNA and protein in the CNS. Overall, we found that the expression levels of *Ube3a*/*UBE3A* are relatively constant among tissues and between eutherian and metatherian mammals, despite different imprinting states. Taken together, these findings suggest that the acquisition of *UBE3A* imprinting in eutherian mammals coincided with increased expression levels, supporting the conclusion that imprinting of *UBE3A* did not evolve as a dosage-regulating mechanism in neurons.

The dosage model of genomic imprinting suggests that imprinting evolved at some loci to reduce the expression levels of the gene by half [50]. For example, the paternal allele of the *Murr1* gene is imprinted in neurons during brain development by the paternally expressed *U2af1*-*rs1* antisense transcript, leading to a substantial reduction in *Murr1* RNA levels in the postnatal brain [57]. Additionally, differential imprinting (i.e., monoallelic and biallelic) of the *Igf2* gene in neurogenic niches of the adult mouse brain has been shown to regulate autocrine and paracrine roles of *Igf2* in a dose-dependent manner [14]. In contrast to these two genes, we show that the levels of *Ube3a*/*UBE3A* RNA and protein are remarkably stable among tissues and species with different imprinting states, which stems from increased expression of the maternal *Ube3a* allele during the acquisition of the imprint. These findings are consistent with previous studies showing relatively constant levels of *Ube3a*/*UBE3A* transcripts during mouse brain development and among human tissues [15, 27] and the notion that dosage compensation may in fact be a common feature of imprinted genes, as suggested previously [3].

Given the importance of UBE3A in the brain and the conservation of the imprint in mouse and human neurons, and perhaps all placental mammals, it is likely that imprinting of *UBE3A* is a functionally relevant process in neurons that is somehow linked to the neuron-specific expression of the PWS polycistronic transcription unit (PWS-PTU). For example, imprinting of *UBE3A* might have evolved as a means to permit simultaneous expression of both *UBE3A* and the PWS-PTU in neurons, similar to that proposed in the complementation model of genomic imprinting [24]. Alternatively, transcription of *UBE3A*-*AS* across *UBE3A* or, perhaps, *UBE3A*-*AS* itself might expand the functional repertoire of UBE3A isoforms (coding and non-coding) expressed in neurons. Unlike most imprinted genes regulated by an antisense transcript, studies in mouse show that *Ube3a*-*AS* inhibits transcriptional elongation of the paternal *Ube3a* allele, resulting in a paternally expressed, 5′-truncated transcript expressed exclusively in neurons [33, 38]. Given recent findings that mouse *Ube3a* expresses a non-coding isoform that is important for synapse development [55], it is tempting to speculate that the 5′-truncated *Ube3a* transcript is in fact a functional transcript rather than just a by-product of the imprinting mechanism.

## Conclusions

Although future studies are needed to resolve the functional significance of imprinting of *UBE3A* in neurons, the findings presented here provide evidence of an evolutionarily constrained, developmentally regulated process that maintains the dosage of UBE3A despite its monoallelic expression in neurons. Understanding the function of imprinting of *UBE3A* is directly relevant to understanding the function of *UBE3A* in the brain, and understanding the imprinted regulation of *UBE3A* is critically important for approaches aimed at reactivating expression of the paternal *UBE3A* allele as a therapy for individuals with Angelman syndrome.

## Methods

### Analysis of Ube3a/UBE3A expression levels in tissues

#### Animals

C57BL/6J mice were obtained from The Jackson Laboratories (http://www.jax.org). The *Ube3a* ^m−/p+^ mouse model was obtained from the laboratory of Dr. Arthur Beaudet (Baylor College of Medicine). *Ube3a* ^m−/p+^ and animals were generated by crossing wild-type C57BL/6J males with *Ube3a* ^m+/p−^ females, and *Ube3a* ^m+/p−^ animals were generated by crossing *Ube3a* ^m+/p−^ males with wild-type C57BL/6J females. Animals were maintained by the Texas A&M University Comparative Medicine Program. All procedures involving animals were approved by the Texas A&M Institutional Animal Care and Use Committee.

#### Quantitative RT-PCR

Total RNA was extracted from tissue samples using the PureLink RNA Mini Kit (Life Technologies, Carlsbad, CA). First-strand cDNA synthesis was performed using: (1) the Superscript III First-Strand Synthesis kit and oligo-dT primers (messenger RNA [mRNA]; Life Technologies) and (2) the High Capacity RNA to cDNA kit and random hexamer primers (total RNA [toRNA]; Life Technologies). Real-time PCR was performed using the TaqMan Gene Expression Master Mix and TaqMan Gene Expression Assays per manufacturer’s protocol (Life Technologies). *Beta*-*2 microglobulin* (TaqMan Assay #Mm00437762_m1) was used as an internal control. TaqMan Assay #Mm00839910_m1 was used to assess *Ube3a* toRNA and mRNA levels. The primer and probe set targets an amplicon of 121 base pairs that spans exons 6 and 7 of *Ube3a* isoforms 1 and 3 and exons 8 and 9 of *Ube3a* isoform 2. The reactions were performed using an ABI 7900HT real-time PCR machine.

#### Statistical analysis

Measurements for inferential statistics were taken using normalized ΔCt values $(2^{{ - \Delta C_{t} }} = 2^{{ - C_{t} \left[ {\text{target}} \right] - C_{t} \left[ {{\text{internal}}\,{\text{control}}} \right]}} )$, as outlined previously [46]. A Shapiro–Wilk goodness-of-fit test was used to test normality of sample distribution. A mixed-effect model was used to examine the effect of tissue on *Ube3a* RNA transcript levels, with sample ID as a random effect to account for repeated measurements of individual. A Tukey HSD multiple comparison test was used for pair-wise comparisons of tissues. The tissues were then grouped as CNS or non-CNS, and a mixed-effect model was used to examine the effect of tissue type on *Ube3a* RNA levels, with sample ID as a random effect. Descriptive statistics consist of ΔΔCt values $\left(2^{{ - \Delta \Delta C_{t} }} = 2^{{ - \left( {C_{t} \left[ {\text{target}} \right] - C_{t} \left[ {{\text{internal}}\,{\text{control}}} \right]} \right) - (C_{t} \left[ {\text{target}} \right] - C_{t} \left[ {{\text{internal}}\,{\text{control}}} \right])}} \right)$; target = *Ube3a*, internal control = *beta*-*2 microglobulin*).

#### Western blot analysis

Total protein was isolated by homogenizing tissue samples with a 1% Nonidet P40/0.01% SDS lysis buffer and protease inhibitors (Roche, Indianapolis, IN). The resulting lysates were mixed 1:1 with Laemmli loading buffer (Bio-Rad, Hercules, CA) and heated to 95 °C for 5 min. The samples were then resolved on a SDS-PAGE gel (7.5%) at 25 V for approximately 12 h and then transferred to nitrocellulose membranes at 100 V for 2 h. To normalize samples, the membranes were treated with Ponceau stain (Sigma-Aldrich) and digitally photographed. The membranes were then blocked in 5% milk in Tris-buffered saline plus Tween 20 (T-TBS) for 1 h at room temperature. Ube3a primary antibody (Additional file 7) was diluted in 2.5% milk/T-TBS and incubated on the membrane for 1 h at room temperature. After three 15 min washes in T-TBS, the secondary antibody (Additional file 7) was diluted 1:2000 in 2.5% milk/T-TBS and incubated on the membrane for 1 h at room temperature. Three 15 min washes in T-TBS were performed before developing with Clarity Western ECL Substrate (Bio-Rad), according to the manufacturer’s protocol. Membranes were imaged using the FluorChem system.

#### Statistical analysis

Digital images of western blot membranes (16bit.tif) were imported into ImageJ, and Ube3a protein levels were quantified using the Gel Analysis feature. Protein levels were quantified (as percentage) and then normalized by the amount of total protein per sample using a Ponceau stain. A Shapiro–Wilk goodness-of-fit test was used to test normality of sample distribution. A linear mixed-effects model was used to examine the association of tissue on Ube3a protein levels, with tissue modeled as a fixed, categorical effect and sample ID modeled as a random effect to account for repeated measurements of individual. A Tukey HSD multiple comparison test was used for pair-wise comparisons of tissues. The tissues were then grouped as CNS or non-CNS, and a linear mixed-effects model was used to examine the effect of tissue type on Ube3a protein levels, with sample ID included as a random effect. Descriptive statistics consist of Ube3a protein levels relative to heart.

#### RNA-sequencing analysis in mouse tissues

Data from Keane et al. [25]: Samples consisted of 76 bp paired-end (PE) libraries generated from total RNA isolated from heart, liver, lung, thymus, and hippocampus of 8-week-old B6D2F1 hybrid mice (C57BL/6 male X DBA female; *n* = 6). FASTQ files were downloaded from the NCBI GEO SRA (accession: ERP000591) and aligned to the mouse reference genome (mm9) using the default settings in TopHat, with the following option: -b2 sensitive.

Normalized RNA steady-state levels of the RefSeq gene annotation were estimated for each sample using Cuffnorm with the following option: -u. The FPKM value of *Ube3a* transcripts for each sample was extracted from the output file and used for descriptive and inferential statistics.

#### Statistical analysis

The parental allelic ratio of *Ube3a* for each tissue was determined using single-nucleotide polymorphisms (SNPs) located in *Ube3a* exon 5 (SNP-1 = chr7:66,527,581) and 8 (SNP-2 = chr7:66,541,539). Raw counts of the 2 SNPs were determined using the CLC Genomics Workbench quality-based variant detection module with the following settings: neighborhood radius = 5; maximum gap and mismatch count = 2, minimum neighborhood quality = 15, minimum central quality = 20, ignore non-specific matches, minimum coverage = 20, minimum variant % = 1. The raw counts of each SNP for each sample and tissue were then used to estimate the fragments per thousand per million (FPKM) values of *Ube3a* transcripts of the parental alleles using the following equations:

$$\begin{aligned} {\text{Maternal}}\,{\text{allele}} & = \left( {\left( {\left( {\sum {\text{SNP}} - 1^{\text{maternal}} } \right)/\left( {\sum {\text{SNP}} - 1^{\text{total}} } \right) + \left( {\sum SNP - 2^{\text{maternal}} } \right)/\left( {\sum {\text{SNP}} - 2^{\text{total}} } \right)} \right)/2} \right) \times {\text{FPKM}} \\ {\text{Paternal}}\,{\text{allele}} & = \left( {\left( {\sum {\text{SNP}} - 1^{\text{paternal}} } \right)/\left( {\sum {\text{SNP}} - 1^{\text{total}} } \right) + \left( {\sum {\text{SNP}} - 2^{\text{paternal}} } \right)/\left( {\sum {\text{SNP}} - 2^{\text{total}} } \right)} \right)/2) \times {\text{FPKM}} \\ \end{aligned}$$

A mixed-effect model was used to examine the effect of tissue and allele on *Ube3a* transcript levels (full factorial), with sample ID as a random effect to account for repeated measurements of individual. A Dunnett’s–Hsu multiple comparison test was used to compare total *Ube3a* transcript levels between hippocampus and each tissue, and a Dunnett’s–Hsu multiple comparison test was used to compare maternal and paternal *Ube3a* transcript levels between hippocampus and each allele of each tissue.

#### RNA-sequencing analysis in human tissues

Data from Fagerberg et al. [12]: Samples consisted of 100 bp PE sequencing libraries generated from mRNA isolated from 12 human tissues. Three samples per tissue were used for the analysis. FASTQ files were downloaded (http://www.ebi.ac.uk/arrayexpress/; accession: E-MTAB-1733) and aligned to the human reference genome (hg19) using the default settings in TopHat. Normalized FPKM values of the RefSeq gene annotation were estimated using Cuffnorm using the default settings and the following option: -u. The FPKM value of *UBE3A* for each sample was extracted from the output file and used for descriptive and inferential statistics.

Data from *GTEx*: The FPKM values and accompanying information were downloaded for *UBE3A* from the GTEx Portal (http://www.gtexportal.org/home/GTEx_Analysis_V4_RNA-seq_RNA-SeQCv1.1.8_gene_rpkm.gct) [10, 53]. The initial data set consisted of 25 different histologic samples derived from 175 individuals (1518 tissue samples). Only tissues for which at least 30 samples were available were included in the inferential analyses (1227 tissue samples from 10 tissues): adipose tissue (*n* = 111), brain (*n* = 296), blood vessels (*n* = 138), esophagus (*n* = 34), heart (*n* = 103), lung (*n* = 113), muscle (*n* = 132), nerve (*n* = 86), skin (*n* = 114), and thyroid (*n* = 100). A total of 291 tissue samples from 14 tissues were excluded. Blood samples were also excluded from inferential analysis.

#### Statistical analysis

A mixed-effect model was used to examine the effect of tissue on *UBE3A* transcript levels, with sample ID as a random effect to account for repeated measurements of individual. For the GTEx data set, tissue and sex were modeled as fixed effects (full factorial), with sample ID as a random effect. A Dunnett’s–Hsu multiple comparison test was used to compare *UBE3A* transcript levels among tissues relative to the brain. Goodness-of-fit of models was assessed by AIC and BIC values and visual inspection of diagnostic residual plots.

### Analysis of allelic Ube3a^YFP^ in neural stem cells and differentiated neurons

#### Animals

The *Ube3a* ^*YFP*^ mouse model was obtained from the laboratory of Dr. Arthur Beaudet (Baylor College of Medicine). *Ube3a* ^*mYFP*/*p*+^ animals were generated by crossing wild-type C57BL/6J males with *Ube3a* ^*m*+/*pYFP*^ females, and *Ube3a* ^*m*+/*pYFP*^ animals were generated by crossing *Ube3a* ^*m*+/*pYFP*^ males with wild-type C57BL/6J females. Wild-type mice consisted of siblings lacking the *Ube3a* ^*YFP*^ allele. PCR to determine the genotypes of mice (i.e., *Ube3a* ^*m*+/*p*+^ or *Ube3a* ^*YFP*^) were performed using methods described previously [11].

#### Neural stem cell cultures

Neural stem cell cultures were established from *Ube3a* ^*m*+/*p*+^, *Ube3a* ^*mYFP*/*p*+^, and *Ube3a* ^*m*+/*pYFP*^ mice using methods described previously [48]. Briefly, the hippocampal formation (HF) was removed from fetuses at embryonic day 17.5 (E17.5), digested using a 10× Trypsin–EDTA solution, triturated into a single cell suspension, and then seeded in neural stem cell (NSC) medium, consisting of DMEM-F12 (Invitrogen, Carlsbad, CA), B-27 supplement (Invitrogen), progesterone, putrescine (Sigma-Aldrich), epidermal growth factor (Sigma-Aldrich), glucose, penicillin/streptomycin (Invitrogen), insulin–transferrin–sodium selenite (Sigma-Aldrich), HEPES, and heparin. The NSC cultures were maintained in humidified incubators at 37 °C and 5% CO_2_. Every 3–4 days, the neurospheres were dissociated with TrypLE (Invitrogen) for 20 min and then resuspended in NSC media.

#### Western blot analysis

Western blot analysis of Ube3a and Ube3a^YFP^ protein levels in NSC cultures was performed using methods described above.

#### Statistical analysis

A mixed-effect model was used to examine the effect of allele (i.e., *Ube3a* or *Ube3a* ^*YFP*^) and parent-of-origin (i.e., maternal or paternal) on Ube3a and Ube3a^YFP^ protein levels (full factorial), with sample ID as a random effect to account for repeated measurements.

#### Neural stem cell differentiation

To differentiate NSC cultures, neurospheres were first dissociated using TrypLE (Invitrogen) following the manufacturer’s protocol. The dissociated cells were then resuspended in neuronal growth media (Neurobasal-A [Invitrogen], B-27 supplement [Invitrogen], and Glutamax [Invitrogen]) and plated on glass coverslips coated with poly-ornithine [Sigma-Aldrich] and laminin [Invitrogen] at a density of 380,000 cells per well in a 12-well, cell-culture plate; the plates were maintained in humidified incubators at 37 °C and 5% CO_2_.

#### Immunofluorescence imaging of Ube3a^YFP^

Immunofluorescence imaging was used to quantify Ube3a^YFP^ protein levels in differentiated neurons as previously described [11]. Briefly, at 1, 4, 8, and 16 days post-differentiation (DPD; day of differentiation = 0 DPD), differentiated cells were washed twice with 1× PBS, fixed in 4% paraformaldehyde in PBS for 15 min, and then washed three times in 1× PBS. The cells were blocked in 0.3% Triton-X100 in PBS (T-PBS) plus 5% goat or donkey serum for 1–2 h at room temperature with gentle agitation. Cells were incubated with the primary antibodies (Additional file 7) for 24 h at 4 °C with gentle agitation. Cells were washed 3 times in 0.1% Tween 20 1× PBS for 15 min each and then incubated with fluorescently labeled secondary antibodies (Additional file 7) for 24 h at 4 °C in the dark. Cells were then washed 4 times in 0.1% Tween 20 1× PBS for 15 min each. Nuclei were labeled using TOPRO-3(Invitrogen) at a dilution of 1:1000 in the third wash. Coverslips were mounted on glass slides with Vectashield (Vector Laboratories, Burlingame, CA) mounting reagent. Confocal images were obtained using a LSM 510 META NLO multiphoton microscope (Zeiss, Oberkochen, Germany). Images of the individual neurons were taken at 43× magnification. Images were imported into ImageJ and converted to the RGB color format. The Plot Profile feature was then used to determine the median gray value of Ube3a^YFP^ in individual neurons. Neurons and astrocytes were identified by immunoreactivity with the Tubb3 and GFAP antibodies, respectively.

#### Statistical analysis

To examine the effect of day on Ube3a^YFP^ protein levels, a linear regression model was used with normalized intensity values of Ube3a^YFP^ as the dependent variable and days post-differentiation (DPD) and allele (i.e., maternal and paternal) as fixed effects. To compare maternal and paternal Ube3a^YFP^ protein levels at each time point, a least square means contrast linear regression model was used with normalized gray values of Ube3a^YFP^ protein levels as the dependent variable and DPD (dummy variable) and allele as fixed effects.

### Analysis of opossum Ube3a/UBE3A expression

#### Animals

For the analysis of UBE3A protein levels, opossum (*Monodelphis domestica*) tissues (cortex, hippocampus, heart, and lung) samples were obtained from a colony at the Comparative Medicine Program facilities at Texas A&M University. All animals were derived from an outbred stock (LL1) that was founded using wild animals captured in Eastern Brazil. All procedures involving animals were approved by the Texas A&M Institutional Animal Care and Use Committee. For the allelic expression analysis of *UBE3A*, opossum fetal brain (cortex) samples were obtained at 13 days post-copulation (d.p.c.) from F1 animals derived from reciprocal crosses of two semi-inbred stocks (LL1 and LL2) [56].

#### RNA-seq analysis

The brain tissues were homogenized in TRI Reagent (Invitrogen) and total RNA was extracted using BCP (1-bromo-2 chloropropane), precipitated with isopropanol, and resuspended in RNase-free water. Potential DNA contamination was removed by both DNase I treatment and DNA removal columns in Qiagen RNeasy Plus Mini kit (Qiagen, CA). RNA-seq libraries were made using the Illumina TruSeq RNA Sample Prep Kit (Illumina Inc., CA), and sequenced on an Illumina HiSeq 2000 instrument (Illumina Inc., CA). Details on RNA-seq data analysis could be found in Wang et al. [56].

#### Statistical analysis

A Pearson’s two-sided Chi-square test of the allelic counts of each SNV was used to determine the whether the allelic ratios of *UBE3A* were equal (Ho: maternal/paternal = 50:50; Ha: maternal/paternal = 50:50).

#### Western blot analysis

Total protein was isolated from adult (21-week-old) opossum tissues (*n* = 4) and adult (8-week-old) mouse cortex (*n* = 4) as described above. For the analysis of UBE3A protein levels among opossum tissues, a single western blot was performed for each animal for all tissues. UBE3A protein levels were estimated and normalized as described above. For the comparison of Ube3a/UBE3A protein levels between opossum and mouse cortex, samples were run on a single western blot, and Ube3a/UBE3A protein levels were normalized and compared as described above.

#### Statistical analysis

To compare UBE3A protein levels among opossum tissues, a mixed-effect model was used with normalized UBE3A protein levels as the dependent variable, tissue as a fixed effect, and sample ID as a random effect to account for repeated measurements of individual. To compare Ube3a/UBE3A protein levels between mouse and opossum cortex, a two-tailed Welsh *t* test was performed.

### Analysis of Ube3a^YFP^ in neurogenic niches of the adult mouse brain

#### Immunofluorescence imaging of Ube3a^YFP^

*Ube3a* ^*m*+/*pYFP*^ and *Ube3a* ^*mYFP*/*p*+^ adult mice (6-week-old) were anesthetized with 0.5–1.0 mL of 20 mg/mL Avertin (Sigma-Aldrich, St. Louis, MO) via intraperitoneal injection. Mice were perfused with ice-cold phosphate-buffered saline (PBS) and 4% paraformaldehyde. Dissected brains were post-fixed in 4% paraformaldehyde solution for approximately 12 h and then cryoprotected in 30% sucrose solution. Fifty µm sections (sagital and coronal) were cut on a cryostat and stored in PBS. For immunostaining, sections were blocked in 0.3% Triton-X100 in PBS (T-PBS) plus 5% serum (goat or donkey) for 1–2 h at room temperature in a humidified chamber with gentle agitation. Primary antibodies (Additional file 7) were incubated with sections for 48 h at 4 °C with gentle agitation. Sections were washed 3 times in 0.1% Tween 20 1× PBS for 15 min each and then incubated with fluorescently labeled secondary antibodies (Additional file 7) for 24 h at 4 °C in the dark with gentle agitation. Sections were washed 4 times in 0.1% Tween 20 plus 1× PBS for 15 min each. Nuclei were labeled by adding TOPRO-3 at 1:1000 dilution in the third wash. Sections were mounted on glass slides with Vectashield (Vector Laboratories, Burlingame, CA) mounting reagent. Confocal images were obtained using a LSM 510 META NLO multiphoton microscope (Zeiss, Oberkochen, Germany). Images were taken using 10× and 43× (oil) objectives. Rostral migratory stream images were taken at 10× magnification, and then images were then stitched together. For resolution of individual cell images in vivo Z-stack images of 8–11 slices were used.

### Analysis of Ube3aYFP in myenteric neurons

#### Immunofluorescence imaging of Ube3a^YFP^

*Ube3a* ^*mYFP*/*p*+^ and *Ube3a* ^*m*+/*pYFP*^ adult mice (6-week-old; *n* = 3/genotype) were processed and immunostained as described above. Confocal images of Auerbach’s ganglia (8–10 per animal) were obtained using a 43× (oil) objective. Images were imported into ImageJ and converted to the RGB color format. The Plot Profile feature was then used to determine the median gray value of Ube3a^YFP^ in individual neurons (RBFOX3 positive) as described above.

#### Statistical analysis

A mixed-effect model was used to examine the effect of allele (maternal and paternal) on Ube3a^YFP^ protein levels, with sample ID as a random effect to account for repeated measurements of neurons within an individual animal.

### Statistics

Inferential analyses were performed using JMP Pro^®^ (version 12). Mixed-effect models were used for analyses involving repeated measures of cells/tissues from an individual animal. The degrees of freedom were calculated using the Kenward–Roger correction.

### Charts

Charts were generated using JMP Pro^®^ (version 12) and formatted in Adobe Illustrator.

## Additional files

**Additional file 1: Table S1.** Pair-wise comparisons of maternal and paternal Ube3a transcript levels.

**Additional file 2: Table S2.** Pair-wise comparisons of UBE3A transcript levels.

**Additional file 3: Table S3.** Pair-wise comparisons of UBE3A transcript levels.

**Additional file 4: Table S4.** RNA-seq analysis of *UBE3A* allelic expression in opossum brain.

**Additional file 5: Figure S1.** Alignment of mouse, opossum, and human Ube3a/UBE3A amino acid sequences. (A) ClustalW alignment of amino acid sequences of human UBE3A isoform 3, mouse Ube3a isoform 2, and opossum UBE3A. (B) Percent identity values of pair-wise comparisons of UBE3A/Ube3a among human, mouse, and opossum. Values represent conservative, semiconservative, non-conserved, and insertion/deletion percent identity.

**Additional file 6: Figure S2.** (A-C) Immunofluorescence images of paternal Ube3a^YFP^ protein expression in the lateral ventricle of adult *Ube3a* ^*+*/*YFP*^ mice. (D) Immunofluorescence image of maternal Ube3a^YFP^ protein expression in the lateral ventricle of adult *Ube3a* ^*YFP*/*+*^ mice. (E) Immunofluorescence images of maternal Ube3a^YFP^ protein expression in the lateral ventricle, subventricular zone, rostral migratory stream, olfactory bulb, and adjacent cortical neurons in *Ube3a* ^*YFP*/*+*^ mice. (F) Immunofluorescence images of maternal Ube3a^YFP^ protein expression in the granular cell layer and subgranular zone of *Ube3a* ^*YFP*/*+*^ mice.

**Additional file 7: Table S5.** List of antibodies used for western blot and immunofluorescence analyses.

## Abbreviations

UBE3A: ubiquitin protein E3A ligase gene
CNS: central nervous system
PTU: polycistronic transcription unit
UBE3A-AS: UBE3A antisense transcript
SNRPN-SNURF: SNRPN upstream reading frame—small nuclear ribonucleoprotein polypeptide N
snoRNA: small nucleolar RNAs
AS: Angelman syndrome
Dup15q: duplication 15q syndrome
PWS: Prader–Willi syndrome
ASD: autism spectrum disorder
Ube3aYFP: Ube3a yellow fluorescent protein
DPD: days post-differentiation
SVZ: subventricular zone
RMS: rostral migratory stream
SGZ: subgranular zone
PSA-NCAM: polysialylated-neural cell adhesion molecule
NES: nestin
DCX: doublecortin
RBFOX3: NA-binding protein, Fox-1 homolog 3
GFAP: glial fibrillary acidic protein
