# Supplementary material for: Genomic imprinting does not reduce the dosage of UBE3A in neurons
Source: Epigenetics Chromatin. 2017 May 15;10:27. doi: 10.1186/s13072-017-0134-4 (PMC5433054; doi:10.1186/s13072-017-0134-4)
Supplement: Supplementary file 1 — Additional file 1: Table S1. Pair-wise comparisons of maternal and paternal Ube3a transcript levels. [file 13072_2017_134_MOESM1_ESM.docx]

| **Additional file 1: Table S1. Pair-wise comparisons of maternal and paternal Ube3a transcript levels.** | | | | | | | | |
| --- | --- | --- | --- | --- | --- | --- | --- | --- |
| **Hippocampus** | **Tissue** | **Allele** | **Diff** | **Std Error** | **Adj Lower 95%** | **Adj Upper 95%** | **t Ratio** | **Adj p value** |
| Maternal | Hippocampus | Paternal | -18.5 | 1.8 | -23.6 | -13.5 | -10.4 | < 0.0001 |
|  | Thymus | Maternal | -8.8 | 1.8 | -13.8 | -3.8 | -4.9 | 0.0001 |
|  | Thymus | Paternal | -10.0 | 1.8 | -15.1 | -5.0 | -5.6 | < 0.0001 |
|  | Liver | Maternal | -8.6 | 1.8 | -13.6 | -3.6 | -4.8 | 0.0002 |
|  | Liver | Paternal | -11.9 | 1.8 | -16.9 | -6.9 | -6.7 | < 0.0001 |
|  | Heart | Maternal | -14.7 | 1.9 | -20.0 | -9.4 | -7.8 | < 0.0001 |
|  | Heart | Paternal | -11.5 | 1.9 | -16.8 | -6.2 | -6.1 | < 0.0001 |
|  | Lung | Maternal | -16.7 | 1.9 | -22.0 | -11.4 | -8.9 | < 0.0001 |
|  | Lung | Paternal | -14.5 | 1.9 | -19.8 | -9.2 | -7.7 | < 0.0001 |
| Paternal | Hippocampus | Maternal | 18.5 | 1.8 | 13.5 | 23.6 | 10.4 | < 0.0001 |
|  | Thymus | Maternal | 9.7 | 1.8 | 4.7 | 14.8 | 5.4 | < 0.0001 |
|  | Thymus | Paternal | 8.5 | 1.8 | 3.5 | 13.5 | 4.8 | 0.0002 |
|  | Liver | Maternal | 9.9 | 1.8 | 4.9 | 15.0 | 5.6 | < 0.0001 |
|  | Liver | Paternal | 6.6 | 1.8 | 1.6 | 11.7 | 3.7 | 0.005 |
|  | Heart | Maternal | 3.9 | 1.9 | -1.4 | 9.2 | 2.1 | 0.3 |
|  | Heart | Paternal | 7.0 | 1.9 | 1.7 | 12.3 | 3.7 | 0.005 |
|  | Lung | Maternal | 1.8 | 1.9 | -3.5 | 7.1 | 1.0 | 0.9 |
|  | Lung | Paternal | 4.0 | 1.9 | -1.3 | 9.3 | 2.1 | 0.2 |
