# Supplementary material for: Genomic imprinting does not reduce the dosage of UBE3A in neurons
Source: Epigenetics Chromatin. 2017 May 15;10:27. doi: 10.1186/s13072-017-0134-4 (PMC5433054; doi:10.1186/s13072-017-0134-4)
Supplement: Supplementary file 2 — Additional file 2: Table S2. Pair-wise comparisons of UBE3A transcript levels. [file 13072_2017_134_MOESM2_ESM.docx]

| **Additional file 2: Table S2. Pair-wise comparisons of UBE3A transcript levels.** | | | | | | | |
| --- | --- | --- | --- | --- | --- | --- | --- |
| **Tissue-1** | **Tissue-2** | **Diff** | **Std Error** | **Adj Lower 95%** | **Ad Upper 95%** | **t Ratio** | **Adj p value** |
| Brain | Nerve | 1.8 | 0.5 | 0.5 | 3.1 | 3.7 | 0.002 |
|  | Adipose Tissue | -0.7 | 0.5 | -2.0 | 0.5 | -1.6 | 0.6 |
|  | Blood Vessel | 1.0 | 0.4 | -0.2 | 2.1 | 2.3 | 0.1 |
|  | Esophagus | 0.7 | 0.7 | -1.3 | 2.7 | 0.9 | 1.0 |
|  | Heart | -0.3 | 0.5 | -1.6 | 1.0 | -0.6 | 1.0 |
|  | Lung | -2.5 | 0.4 | -3.7 | -1.3 | -5.6 | < 0.0001 |
|  | Muscle | 15.3 | 0.4 | 14.1 | 16.4 | 36.7 | < 0.0001 |
|  | Skin | -1.2 | 0.4 | -2.4 | 0.0 | -2.6 | 0.06 |
|  | Thyroid | 2.0 | 0.5 | 0.7 | 3.3 | 4.3 | 0.0001 |
