# Supplementary material for: Genomic imprinting does not reduce the dosage of UBE3A in neurons
Source: Epigenetics Chromatin. 2017 May 15;10:27. doi: 10.1186/s13072-017-0134-4 (PMC5433054; doi:10.1186/s13072-017-0134-4)
Supplement: Supplementary file 3 — Additional file 3: Table S3. Pair-wise comparisons of UBE3A transcript levels. [file 13072_2017_134_MOESM3_ESM.docx]

| **Additional file 3: Table S3. Pairwise-comparisons of UBE3A transcript levels.** | | | | | | | |
| --- | --- | --- | --- | --- | --- | --- | --- |
| **Tissue-1** | **Tissue-2** | **Diff** | **Std Error** | **Adj Lower 95%** | **Adj Upper 95%** | **t Ratio** | **Adj p value** |
| Cortex | Bonemarrow | -11.2 | 3.7 | -22.9 | 0.6 | -3.0 | 0.07 |
|  | Esophagus | -5.6 | 3.8 | -17.7 | 6.4 | -1.5 | 0.7 |
|  | Gallbladder | -12.8 | 3.8 | -24.9 | -0.8 | -3.4 | 0.03 |
|  | Heart | 3.9 | 3.7 | -7.8 | 15.7 | 1.1 | 0.9 |
|  | Kidney | -8.3 | 3.5 | -19.3 | 2.6 | -2.4 | 0.2 |
|  | Liver | -0.4 | 3.5 | -11.3 | 10.6 | -0.1 | 1.0 |
|  | Lung | -13.3 | 3.8 | -25.4 | -1.2 | -3.5 | 0.03 |
|  | Skin | -4.4 | 3.8 | -16.4 | 7.7 | -1.1 | 0.9 |
|  | Spleen | -14.0 | 3.1 | -23.7 | -4.4 | -4.6 | 0.003 |
|  | Stomach | -11.9 | 3.2 | -22.1 | -1.7 | -3.7 | 0.02 |
|  | Thyroid | -2.6 | 3.8 | -14.7 | 9.4 | -0.7 | 1.0 |
