# Supplementary material for: Genomic imprinting does not reduce the dosage of UBE3A in neurons
Source: Epigenetics Chromatin. 2017 May 15;10:27. doi: 10.1186/s13072-017-0134-4 (PMC5433054; doi:10.1186/s13072-017-0134-4)
Supplement: Supplementary file 4 — Additional file 4: Table S4. RNA-seq analysis of UBE3A allelic expression in opossum brain. [file 13072_2017_134_MOESM4_ESM.docx]

| **Additional file 4: Table S4. RNA-seq analysis of *UBE3A* allelic expression in opossum brain** | | | | | | | | |
| --- | --- | --- | --- | --- | --- | --- | --- | --- |
| **Animal** | **Cross**  **Female X Male** | **SNV** | **chr7 coordinates** | **Reference Allele** | **Alternate Allele** | **SNV location** | **Count** | **Allele** |
| A | LL1XLL2 | 4 | 58706103 | A | T | UTR3 | 25 | Maternal |
| B | LL1XLL2 | 4 | 58706103 | A | T | UTR3 | 30 | Maternal |
| C | LL2XLL1 | 4 | 58706103 | A | T | UTR3 | NI | Maternal |
| D | LL2XLL1 | 4 | 58706103 | A | T | UTR3 | NI | Maternal |
| A | LL1XLL2 | 4 | 58706103 | A | T | UTR3 | 49 | Paternal |
| B | LL1XLL2 | 4 | 58706103 | A | T | UTR3 | 24 | Paternal |
| C | LL2XLL1 | 4 | 58706103 | A | T | UTR3 | NI | Paternal |
| D | LL2XLL1 | 4 | 58706103 | A | T | UTR3 | NI | Paternal |
| A | LL1XLL2 | 3 | 58699719 | A | G | Exon | 39 | Maternal |
| B | LL1XLL2 | 3 | 58699719 | A | G | Exon | 20 | Maternal |
| C | LL2XLL1 | 3 | 58699719 | A | G | Exon | 39 | Maternal |
| D | LL2XLL1 | 3 | 58699719 | A | G | Exon | 43 | Maternal |
| A | LL1XLL2 | 3 | 58699719 | A | G | Exon | 49 | Paternal |
| B | LL1XLL2 | 3 | 58699719 | A | G | Exon | 18 | Paternal |
| C | LL2XLL1 | 3 | 58699719 | A | G | Exon | 33 | Paternal |
| D | LL2XLL1 | 3 | 58699719 | A | G | Exon | 43 | Paternal |
| A | LL1XLL2 | 2 | 58597638 | C | T | UTR5 | 84 | Maternal |
| B | LL1XLL2 | 2 | 58597638 | C | T | UTR5 | 68 | Maternal |
| C | LL2XLL1 | 2 | 58597638 | C | T | UTR5 | 66 | Maternal |
| D | LL2XLL1 | 2 | 58597638 | C | T | UTR5 | 54 | Maternal |
| A | LL1XLL2 | 2 | 58597638 | C | T | UTR5 | 89 | Paternal |
| B | LL1XLL2 | 2 | 58597638 | C | T | UTR5 | 74 | Paternal |
| C | LL2XLL1 | 2 | 58597638 | C | T | UTR5 | 58 | Paternal |
| D | LL2XLL1 | 2 | 58597638 | C | T | UTR5 | 54 | Paternal |
| A | LL1XLL2 | 1 | 58597560 | C | G | UTR5 | 79 | Maternal |
| B | LL1XLL2 | 1 | 58597560 | C | G | UTR5 | 69 | Maternal |
| C | LL2XLL1 | 1 | 58597560 | C | G | UTR5 | 59 | Maternal |
| D | LL2XLL1 | 1 | 58597560 | C | G | UTR5 | 68 | Maternal |
| A | LL1XLL2 | 1 | 58597560 | C | G | UTR5 | 96 | Paternal |
| B | LL1XLL2 | 1 | 58597560 | C | G | UTR5 | 63 | Paternal |
| C | LL2XLL1 | 1 | 58597560 | C | G | UTR5 | 58 | Paternal |
| D | LL2XLL1 | 1 | 58597560 | C | G | UTR5 | 68 | Paternal |
| Abbreviations: NI, SNV count not informative. | | | | | | | | |
