# Supplementary figures and images for: Genomic imprinting does not reduce the dosage of UBE3A in neurons
Source: Epigenetics Chromatin. 2017 May 15;10:27. doi: 10.1186/s13072-017-0134-4 (PMC5433054; doi:10.1186/s13072-017-0134-4)

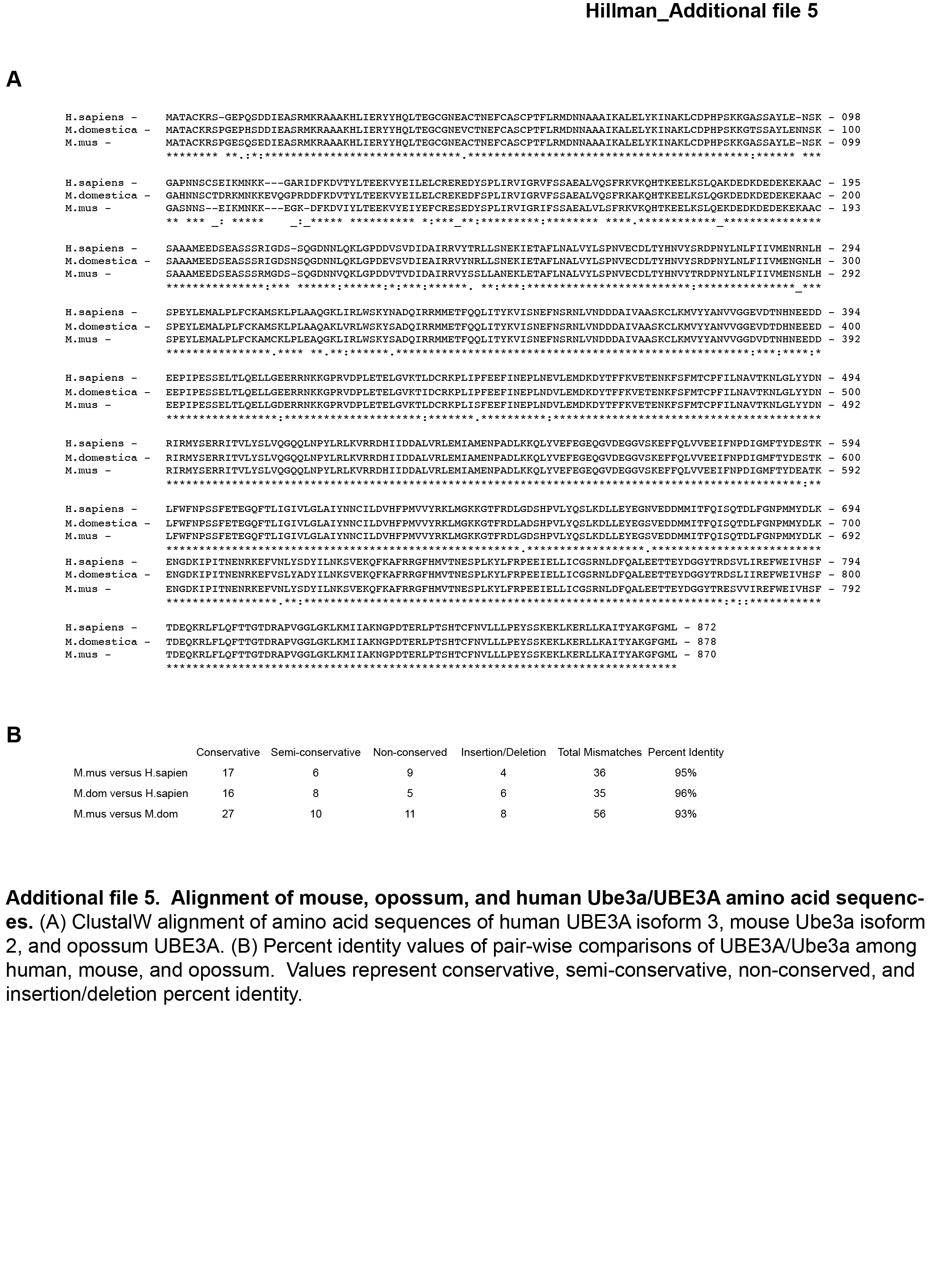

Supplement: Supplementary file 5 — Additional file 5: Figure S1. Alignment of mouse, opossum, and human Ube3a/UBE3A amino acid sequences. (A) ClustalW alignment of amino acid sequences of human UBE3A isoform 3, mouse Ube3a isoform 2, and opossum UBE3A. (B) Percent identity values of pair-wise comparisons of UBE3A/Ube3a among human, mouse, and opossum. Values represent conservative, semiconservative, non-conserved, and insertion/deletion percent identity. [file 13072_2017_134_MOESM5_ESM.png]

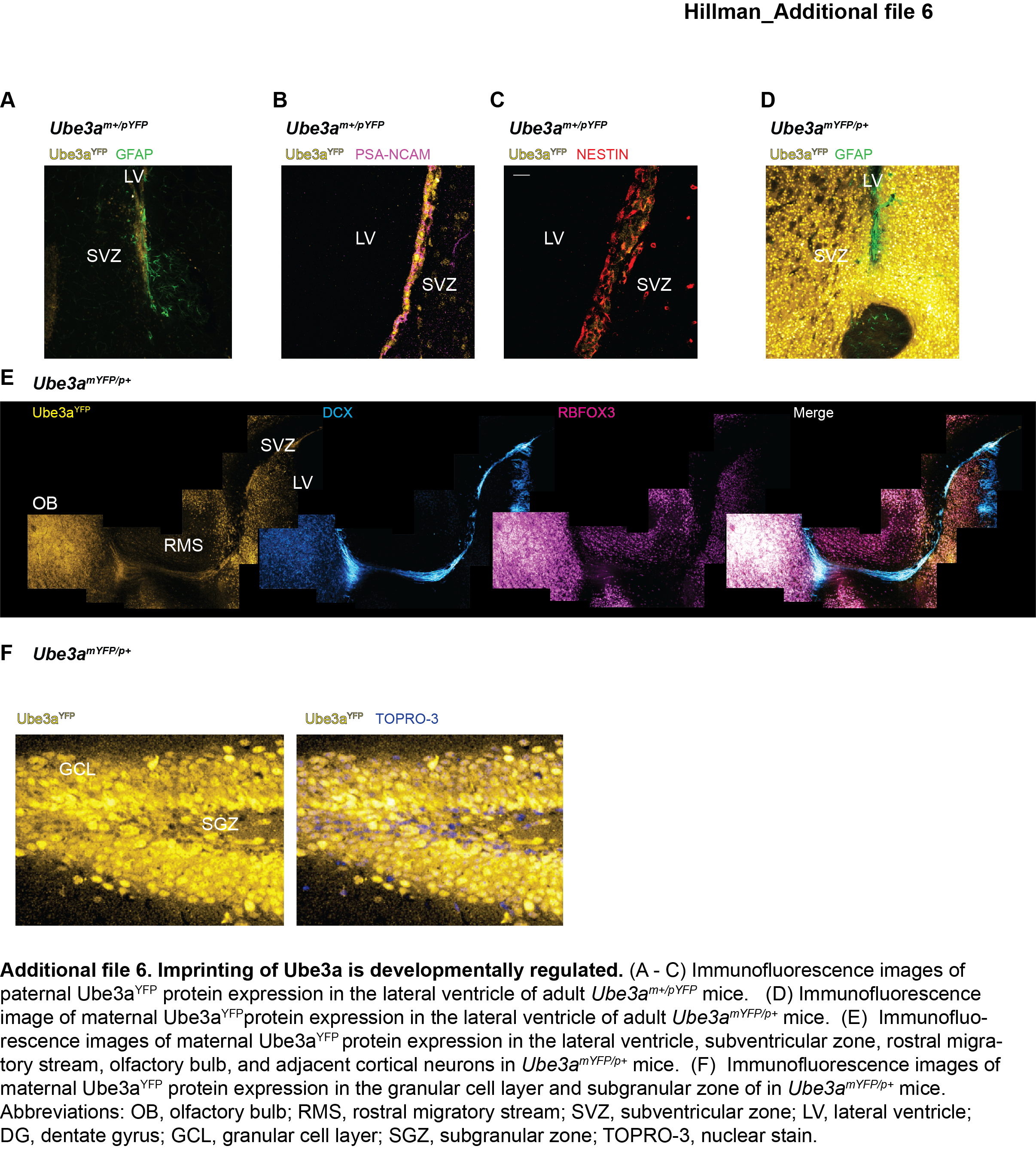

Supplement: Supplementary file 6 — Additional file 6: Figure S2. (A-C) Immunofluorescence images of paternal Ube3aYFP protein expression in the lateral ventricle of adult Ube3a +/YFP mice. (D) Immunofluorescence image of maternal Ube3aYFP protein expression in the lateral ventricle of adult Ube3a YFP/+ mice. (E) Immunofluorescence images of maternal Ube3aYFP protein expression in the lateral ventricle, subventricular zone, rostral migratory stream, olfactory bulb, and adjacent cortical neurons in Ube3a YFP/+ mice. (F) Immunofluorescence images of maternal Ube3aYFP protein expression in the granular cell layer and subgranular zone of Ube3a YFP/+ mice. [file 13072_2017_134_MOESM6_ESM.png]
