# Supplementary material for: Genomic imprinting does not reduce the dosage of UBE3A in neurons
Source: Epigenetics Chromatin. 2017 May 15;10:27. doi: 10.1186/s13072-017-0134-4 (PMC5433054; doi:10.1186/s13072-017-0134-4)
Supplement: Supplementary file 7 — Additional file 7: Table S5. List of antibodies used for western blot and immunofluorescence analyses. [file 13072_2017_134_MOESM7_ESM.docx]

| **Additional file 7: Table S5. List of antibodies used for western blot and immunofluorescence analyses.** | | | | | | |
| --- | --- | --- | --- | --- | --- | --- |
| **Antibody** | **Host** | **Catalog Number** | **Vendor** | **Location** | **Dilution** | **Application** |
| anti-GFP | Rabbit | NB 600-308 | Novus Biologicals | Littleton, CO | 1:1000 | IF |
| anti-GFAP | Mouse | MAB360 | Millipore | Billerica, MA | 1:250 | IF |
| anti-NeuN | Mouse | 05-557 | Millipore | Billerica, MA | 1:200 | IF |
| anti-DCx | Goat | SC-8066 | Santa Cruz Biotechnology Inc. | Santa Cruz, CA | 1:500 | IF |
| anti-PSA-NCAM | Mouse | MAB5324 | Millipore | Billerica, MA | 1:250 | IF |
| anti-Tubb3 | Mouse | T5076 | Sigma-Aldrich | St. Louis, MO | 1:250 | IF |
| anti-E6AP | Mouse | E8655 | Sigma-Aldrich | St. Louis, MO | 1:500 | WB |
| anti-Ube3a | Mouse | 611416 | BD Biosciences | San Jose, CA | 1:1000 | IF, WB |
| anti-Mouse HRP | Goat | 315-035-003 | Jackson ImmunoResearch | West Grove, PA | 1:2000 | WB |
| anti-Mouse 488 | Goat | 115-545-166 | Jackson ImmunoResearch | West Grove, PA | 1:250 | IF |
| anti-Mouse Cy3 | Goat | 115-165-166 | Jackson ImmunoResearch | West Grove, PA | 1:250 | IF |
| anti-Rabbit 488 | Goat | 111-545-144 | Jackson ImmunoResearch | West Grove, PA | 1:250 | IF |
| anti-Rabbit Cy3 | Goat | 111-165-144 | Jackson ImmunoResearch | West Grove, PA | 1:250 | IF |
| anti-Goat 647 | Donkey | 705-605-147 | Jackson ImmunoResearch | West Grove, PA | 1:250 | IF |
| anti-Rabbit 488 | Donkey | 711-545-152 | Jackson ImmunoResearch | West Grove, PA | 1:250 | IF |
| anti-Mouse Cy3 | Donkey | 715-165-151 | Jackson ImmunoResearch | West Grove, PA | 1:250 | IF |
| Abbreviations: IF, immunofluorescence; WB, western blot | | | | | | |
